# Assessing mold growth severity on various textiles: comparative analysis of inoculum densities and fungal strains

**DOI:** 10.1093/sumbio/qvae035

**Published:** 2024-12-18

**Authors:** Ann Hammad, Kendall Beaugrand, Joseph A Ross, Nick Allan, Martin Berman

**Affiliations:** Microbiology Department, Chinook Contract Research Inc., Airdrie AB T4A 0C3, Canada; Microbiology Department, Chinook Contract Research Inc., Airdrie AB T4A 0C3, Canada; Microbiology Department, Chinook Contract Research Inc., Airdrie AB T4A 0C3, Canada; Microbiology Department, Chinook Contract Research Inc., Airdrie AB T4A 0C3, Canada; Micro-Pak Ltd., 9 Canton Road, Tsim Sha Tsui, Kowloon, Hong Kong

**Keywords:** overseas shipping, textile mold, visible growth

## Abstract

Mold growth on textiles during overseas shipping poses significant economic and health risks, necessitating a comprehensive understanding of fungal contamination dynamics. This study evaluated textile susceptibility to fungal contamination during transit by examining growth patterns, onset times, and determining minimum fungal densities for visible mold growth. Inoculations of six fungal strains (individual and pooled) were conducted at inoculum densities ranging from 10^2^ to 10^6^ spores/ml on various textile materials. The study comprised two phases: pilot growth (PG, with glucose) and Simulated Shipping Conditions (SSC, without glucose). PG yielded mycelial mats on textiles for all fungal strains except *Penicillium citrinum* and *Stachybotrys chartarum*. SSC exhibited variable susceptibilities, with cotton and suede highly susceptible and polyester, imitation suede, and polyurethane notably resistant to mycelial growth. Minimum fungal densities for visible mold growth were established for each textile based on SSC. The findings reveal distinct fungal susceptibilities among textiles, influenced by material composition and structure. Consistent growth on certain textiles at lower fungal densities highlights the need for targeted preventive measures. This research provides crucial insights for the manufacturing and shipping industries, guiding the development of effective mold prevention strategies and establishment of contamination limits during overseas transport.

**Sustainability Statement**

This research supports two key UN Sustainable Development Goals: SDG3 (good health and well-being) and SDG12 (responsible consumption and production). Our study on mold growth in textiles during shipping significantly contributes to SDG3 by addressing a critical public health concern. Mold exposure can lead to various health issues, including respiratory problems, allergic reactions, and other complications, particularly affecting vulnerable populations. By identifying minimum fungal densities for visible growth and textile susceptibilities, we enable the implementation of targeted preventive measures that can substantially reduce health risks associated with mold-contaminated textiles. This research also has implications for occupational health in the textile and shipping industries, potentially improving working conditions and reducing workers’ exposure to harmful mold spores. Furthermore, our findings contribute to safer consumer products and healthier indoor environments, as mold-free textiles promote better air quality in homes and workplaces.

Simultaneously, this study advances SDG12 by promoting responsible production practices in the global textile industry. Our research can significantly reduce product waste and economic losses, potentially decreasing the use of harmful anti-fungal treatments. By improving textile resilience during transit, we contribute to more efficient resource use and reduced environmental impact, aligning with sustainable consumption and production principles. This dual focus on health and responsible production demonstrates the far-reaching implications of our research in promoting global sustainability and well-being.

## Introduction

Microbial contamination profoundly impacts textiles during transit, causing color and fiber degradation, unpleasant odors, and increased potential health risks (Szostak-Kotowa 2004). Traditional fibers lack resistance to microbial accumulation, especially cellulosic fibers like cotton, linen, and rayon. Proteins in wool and silk can also fall victim to mold, and even synthetic fibers such as nylon and polyester may succumb, particularly if soiled or possessing finishes that serve as a carbon source (Szostak-Kotowa 2004).

Mold proliferation becomes a concern during the extended transit of imported products, particularly from regions in the Far East experiencing heightened humidity levels during the rainy seasons or El Niño events (Erren, P; Owner of Erren Recondition B.V., Reconditioning Professional, Product Saver, Rework Specialist. Interview 31 January 2024). In some cases, manufacturers may not adhere to warehouse regulations, potentially resulting in trapped moisture and mold propagation. The risk is particularly pronounced for footwear and apparel, given their composition of organic fibers that provide an ideal substrate for mold growth. Even a minor instance of mold development has the potential to escalate, affecting an entire shipment by the time it reaches its destination.

Shoes have been found to be more prone to mold contamination and growth than apparel due to their complex structure, which includes various organic textiles and solvent or water-based materials. Additionally, the enclosed spaces within shoes create an environment conducive to moisture retention, especially when exposed to diverse climates during shipping. The intricate patterns and layers of materials in shoes offer numerous crevices where moisture can accumulate, fostering conditions suitable for mold proliferation. Global shoe production is estimated at around 22 billion units per year (Anderson 2023), while apparel production is estimated to be eight to ten times greater.

Quantifying the impact of mold damage proves to be an arduous task due to the multitude of contributing variables (Neuner, E; President of NuShoe, Inc. Interview 02 February 2024). Merchandise arriving at the destination with mold damage can result in unexpected remediation costs, delays in deliveries, destruction of damaged merchandise, missed advertisements, loss of brand reputation, and claims of health hazards. (Erren, P; Owner of Erren Recondition B.V., Reconditioning Professional, Product Saver, Rework Specialist. Interview 31 January 2024); (Neuner, E; President of NuShoe, Inc. Interview 02 February 2024) Moreover, even a small percentage of a delivery arriving with mold can often mean a complete item-by-item inspection or in many cases a total rejection of the consignment and a major financial loss.

Despite the omnipresence of mold spores in the air, their ability to proliferate hinges on conducive environmental conditions. Mold growth is facilitated by relative humidity (RH) levels ranging from 65% to 100%, warm temperatures, and inadequate air circulation (Michalski 1993). Maintaining RH below 60% and ensuring proper air circulation reduces the likelihood of mold growth (Guild 2004, Ferreira 2018). Mold prevention in overseas shipping containers is challenging due to the interplay between external conditions—such as temperature, humidity, and material reactivity—and microbial growth (Szostak-Kotowa 2004). Factors like condensation and temperature fluctuations within containers create an environment conducive to mold growth, making it essential to explore alternative strategies and better understand mold growth dynamics and minimal contamination densities for effective prevention that can be applied to overseas shipping conditions.

In the distribution environment, packages encounter diverse environmental conditions, with shipments consisting of three stages. The first stage involves container loading, land transport, and harbor storage until ship loading. The second stage occurs at sea aboard a ship. The final stage begins with unloading, followed by customs procedures, land transport, and storage (Weiskircher 2008). The International Safe Transit Association (ISTA) recognizes eight environmental conditions with temperature ranges of −29°C–60°C and RH ranges of 15%−85% RH (ISTA 2018). Xerox Corporation validated ISTA ranges in a study of ocean containers in 160 shipments along three routes between April 2004 and January 2006 (Leinberger 2006). Findings showed exposure to temperature ranges of −29°C–57°C, aligning closely with the ISTA. Containers experienced RH ranges from 32% to 96% RH, overlapping the ISTA range. Daily temperature differences on land during shipping’s first and final stages exhibited extreme temperature swings of 22°C. Container positioning on the ship’s deck influenced temperature variations, where exposed containers experienced larger daily variations compared to the more temperature-consistent shielded containers. Furthermore, the research explored the effects of position and container contents on humidity. Porous packaging materials, like corrugated boxes, and porous goods, like apparel, slowly absorb moisture from the air at lower temperatures, predominantly during night-time hours (Leinberger 2006).

Despite comprehensive studies on textile biodegradation in soil and landfills (Szostak-Kotowa 2004, Li 2010 et al. 2010, Arshad 2014 et al. 2014, Milošević 2017, Jamee 2019, Egan 2022, Sunidhi 2023) and microbial biodegradation of ancient textiles (Michalski 1993, Gutarowska et al. 2017, Geweely 2023), a significant gap exists in understanding the impact of overseas container shipping conditions on mold growth on textiles. The influence of extended transit periods, varying environmental conditions, and the potential for mold proliferation during shipping has yet to be fully explored. The present study simulates mold growth on different textile materials under conditions representative of overseas container shipping. Notably, this study pioneers an investigation into the minimum starting mold density on textiles that can induce visible damage, providing invaluable insights for consumer goods manufacturers. With only a few companies budgeting for mold damage and reconditioning, this research aims to equip consumer goods manufacturers with a predictive tool to assess the likelihood of mold growth during shipping, enabling them to make informed decisions and mitigate economic losses. Understanding biodeterioration dynamics under specific shipping conditions contributes to the development of effective strategies for protecting raw materials and finished products, enhancing textile resilience in transit.

## Materials and methods

### Strains and growth conditions

The fungal strains used in this study were selected to represent a diverse range of genera commonly associated with mold growth on textiles and in indoor environments. *Aspergillus niger* (ATCC 6275), *Penicillium citrinum* (ATCC 36382), *Chaetomium globosum* (ATCC 6205), *Stachybotrys chartarum* (ATCC 16026), *Trichoderma reesei* (ATCC 13631), and *Cladosporium sphaerospermum* (ATCC 11289) were chosen for their relevance to the textile industry, indoor air quality concerns, and potential for biodegradation of cellulose-based materials. These strains exhibit varied growth characteristics and ecological niches, allowing for a comprehensive assessment of mold severity across different textile types. Potato dextrose agar plates (PDA; PP85, Dalynn Biologicals Inc., Calgary, AB, Canada) were used to restore the frozen stocks of each microorganism, except for *Ch. globosum*, by spread plating their primary cultures and incubating at 28°C. *Aspergillus niger, P. citrinum*, and *T. reesei* were incubated for 14 days, whereas *S. chartarum* and *Cl. sphaerospermum* were incubated for 21 days. Spores were harvested by adding 7–8 ml of sterile distilled water (Culligan, Calgary, AB, Canada) to a plate with abundant growth and gently scraping the surface of the culture with a sterile L-shaped spreader (VWR International, Mississauga, Ontario, Canada) to liberate the spores without detaching mycelial fragments. The spore suspension was gently decanted into a sterile flask with 20 sterile glass beads (VWR International, Mississauga, Ontario, Canada). An additional 100 ± 1 ml sterile water was used for *A. niger* and *T. reesei* due to thick spore suspensions. Flasks were shaken to bring the spores into suspension. The spore suspension was then filtered through 4 thin layers of sterile medical gauze (Dukal Corp., Ronkonkoma, NY, USA) into a sterile flask to remove mycelial fragments. Spore counts were carried out with a hemocytometer (American Optical Corp., Buffalo, NY, USA) and spore suspensions were diluted to desired densities.

Per Test Method 30 (TM30) of the American Association of Textile Chemists and Colorists (AATCC) (AATCC 2021) Test II, Mineral Salt Agar (MSA) plates without glucose (MSA-glu; PM51, Dalynn Biologicals Inc., Calgary, AB, Canada) with a disc of sterile filter paper (Whatman Grade 1, Cytvia, Marlborough, MA, USA) were used to restore the *Ch. globosum* frozen stock by inoculating the filter paper with 0.5 ml and incubating at 28°C for 18 days for abundant growth. Spores were harvested as described above by combining the filter papers from the MSA plates in a sterile flask containing 100 ± 2 ml sterile distilled water and 20 sterile glass beads (VWR International, Mississauga, Ontario, Canada).

### Textile materials and swatch preparation

Cotton (Testex GmbH & Co., Bad Münstereifel, Germany), genuine leather, genuine suede, imitation suede, polyurethane, and polyester (locally sourced, Zhongshan, China) materials were used in this study. None of the sourced materials had undergone any anti-microbial treatment. Square swatches 3.37 cm^2^ ± 0.45 cm^2^ were prepared for each material, per AATCC TM30 recommendation, and sterilized with ethylene oxide (Anprolene Sterilizers, Haw River, NC, USA) for 12 h followed by 2 h of aeration. Filter paper (Whatman Grade 1, Cytiva, Marlborough, MA, USA) swatches were used for growth and negative controls. Swatches were pre-dampened in 0.05% Tween-80, a non-ionic wetting agent, before being placed on the agar surface.

### Pilot growth

Spore inoculums were prepared for each fungal strain by diluting spore suspensions to approximately 8.0 × 10^5^ to 1.4 × 10^6^ CFU/ml in sterile distilled water, as recommended in AATCC TM30. A pooled mix was prepared by mixing equal volumes of spore inoculum from each of the six fungal strains. Viability checks were performed for each fungal strain by spread plating 100 µl on MSA plates with 3% ± 0.1% glucose (MSA + glu; PM52, Dalynn Biologicals Inc., Calgary, AB, Canada) in duplicates and incubating at 35°C ± 2°C at 65–90% RH for 1–2 days. Spore inoculum densities were confirmed with a 0.1 mm deep Bright Line hemocytometer (American Optical Corporation, Buffalo, NY, USA). For each fungal strain and the pooled mix, 0.5 ± 0.01 ml of inoculum was spread plated onto MSA + glu. Negative controls were spread-plated with 0.5 ± 0.01 ml sterile distilled water. Sterile textile swatches, growth control filter papers, and negative control filter papers were pre-wetted in 0.05% Tween-80 (MP Biomedicals, Solon, OH, USA), and placed onto the agar surface. Each swatch and growth control filter paper was then inoculated with 0.2 ± 0.01 ml of the corresponding inoculum and allowed to dry overnight at room temperature. Negative controls were spread-plated with 0.2 ± 0.01 ml sterile distilled water. Each of the six textile materials and filter paper was inoculated with each of the six fungal strains and the pooled mix in triplicates. All plates were incubated at 35°C ± 2°C at 65%–90% RH for 7 days. Swatches were visually inspected using a phase contrast inverted microscope (Nikon, Japan) at 7–50× every day for up to 7 days.

### Simulated shipping conditions growth

Spore inoculums were prepared for each fungal strain by serially diluting spore suspensions to approximately 10^6^, 10^5^, 10^4^, 10^3^, and 10^2^ CFU/ml. Pooled mixes were prepared by mixing equal volumes of each dilution from each of the six fungal strains.

Viability checks were performed for each fungal strain at each inoculum density by spread plating 100 µl on MSA + glu in duplicates and incubated at 35°C ± 2°C at 65%–90% RH for 1–2 days. Spore inoculum densities for each dilution were confirmed with a hemocytometer, where possible (thus reported as spores/ml), and confirmed by serially diluting in 1× PBS (Cytiva, HyClone Laboratories, West Jordan, UT, USA) and spot-plating onto PDA plates. Where counts were not performed on the hemocytometer, viable CFU/ml were reported instead. Initial runs were carried out with 10^6^, 10^5^, and 10^4^ CFU/ml inoculums for all strains and pooled mix on textiles that showed successful growth in pilot growth (PG) runs. Inoculums of 10^3^ and 10^2^ CFU/ml were tested only if growth was observed with 10^4^ CFU/ml.

For each fungal strain and the pooled mix, 0.5 ± 0.01 ml of inoculum was spread plated onto MSA plates without glucose (MSA-glu; PM52, Dalynn Biologicals Inc., Calgary, AB, Canada). Negative controls were spread-plated with 0.5 ± 0.01 ml sterile distilled water. Sterile textile swatches, growth control filter papers, and negative control filter papers were pre-wetted in 0.05% Tween-80 and placed onto the agar surface. Each swatch and growth control filter paper was then inoculated with 0.2 ± 0.01 ml of the corresponding inoculum and allowed to dry overnight at room temperature. Negative controls were spread-plated with 0.2 ± 0.01 ml sterile distilled water. Each of the six textile materials and filter paper was inoculated with each of the six fungal strains and the pooled mix at one of the five spore densities tested in triplicates. All plates were incubated at 35°C ± 2°C at 65%–90% RH for 6 weeks, conditions chosen to simulate worst-case scenario for extended overseas shipping and storage in warm climates.

Each plate and swatch received 0.5 and 0.2 ml of inoculum, respectively, for a total inoculum volume of 0.7 ml. Final inoculum densities were therefore approximately: 10^2^ CFU/ml, 10^3^ CFU/ml, 10^4^ spores/ml, 10^5^ spores/ml, and 10^6^ spores/ml.

Swatches with 10^6^, 10^5^, and 10^4^ CFU/ml inoculums were visually inspected using a dissecting microscope at 7–50× every other day for the first week followed by once a week for up to 6 weeks. Swatches with 10^3^ and 10^2^ CFU/ml inoculums were visually inspected using an inverted microscope at 7–50× every week for up to 6 weeks.

### Interpretation and evaluation of results

A visual assessment of observed growth was carried out for all SSC swatches at each inspection time point. The Visual Mold Assessment Score (VMAS) was based on a grading scheme where 0 = no growth, 1 = microscopic growth, and 2 = macroscopic growth. The mean VMAS was calculated for each single strain and pooled mix or textile material at each inoculum density and incubation timepoint. Heat maps of the mean VMAS by inoculated fungal strain and by inoculated textile material were generated using GraphPad Prism version 10.1.2.

## Results

### Pilot growth

**Figure 1.**
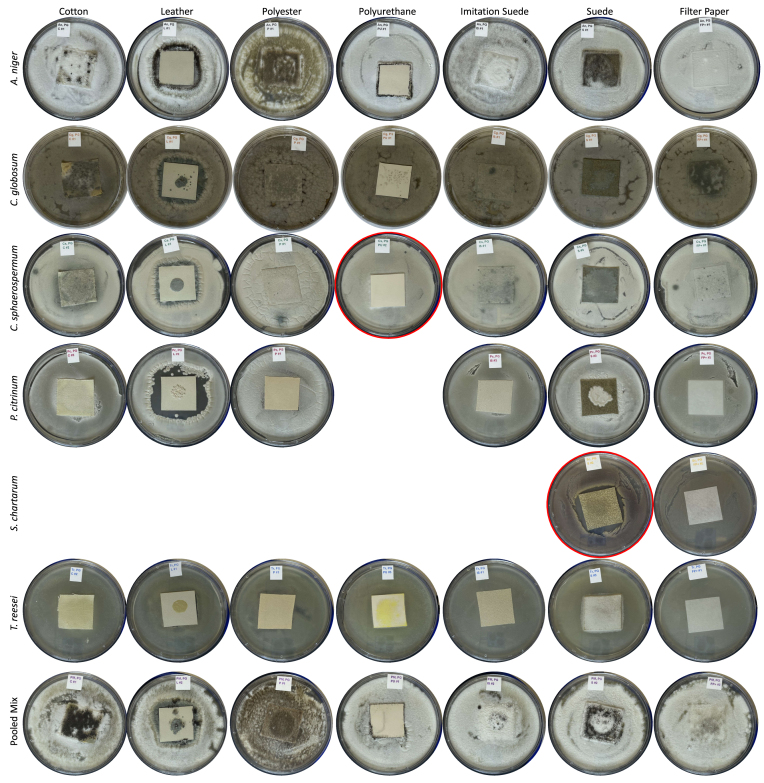
Representative images depicting growth on PG plates at day 7 of incubation, except for *S. chartarum*, which was observed on day 14. MSA with glucose served as the growth medium, where both plates and textile swatches were inoculated with *A. niger, Ch. globosum, Cl. sphaerospermum, P. citrinum, S. chartarum, T. reesei*, or a pooled mix of these fungal strains. Each fungal strain and the pooled mixed culture were inoculated onto cotton, leather, polyester, polyurethane, imitation suede, suede, and filter paper, each in triplicate (*n* = 3). Plates featuring microscopic growth are indicated with highlighted borders.

Negative controls exhibited no visible or microscopic growth throughout the entire 7-day incubation period, confirming the absence of contamination. Conversely, growth controls displayed visible growth as early as day one of incubation. The diluted spore suspensions, ranging from 9.13 × 10^5^ to 1.39 × 10^6^ spores/ml, demonstrated mycelial mat growth surrounding the textile swatches for all tested fungal strains. Notably, visible growth was observed on all textile swatches for each strain and the pooled mix, except for *P. citrinum* and *S. chartarum* (Fig. 1). *Penicillium citrinum* did not exhibit growth on polyurethane, while *S. chartarum* growth was restricted to suede.

Zones of inhibition, denoting growth-free areas, were evident around leather swatches for *A. niger, Ch. globosum, Cl. sphaerospermum, P. citrinum, S. chartarum*, and the pooled mix (Figs S1−S5, S7). Additionally, suede swatches for *S. chartarum* displayed zones of inhibition (Fig. 1, Fig. S5). Remarkably, *A. niger, Ch. globosum, Cl. sphaerospermum*, and the pooled mix exhibited spore growth extending into the zones of inhibition as incubation progressed (Fig. 1, Figs S1−S3, S7).

Upon closer examination, *A. niger, Ch. globosum, T. reesei*, and the pooled mix exhibited macroscopic growth on all inoculated textile swatches (Fig. 1, Figs S1, S2, and S6). *Cladosporium sphaerospermum* exhibited macroscopic growth on all materials except for polyurethane, where growth was microscopic (Fig. 1, Fig. S3). *Penicillium citrinum* showed macroscopic growth on all materials, excluding polyurethane, where no growth was observed (Fig. 1, Fig. S4). In contrast, *S. chartarum* exhibited macroscopic growth solely on filter paper after seven days of incubation, with microscopic growth observed on suede only after 14 days (Fig. 1, Fig. S5).

### Simulated shipping conditions growth

To build on the insights gained from the Pilot Growth, a simulated shipping condition (SSC) test was conducted. Initial high inoculum density (10^6^, 10^5^, and 10^4^ CFU/ml) runs were carried out for *A. niger, Ch. globosum, T. reesei*, and *Cl. sphaerospermum* on all textiles. *Penicillium citrinum* inoculations were performed for all textiles except polyurethane, and *S. chartarum* textile inoculations were performed exclusively on suede. Where growth was observed for the 10^4^ CFU/ml inoculum, low inoculum density runs (10^3^ and 10^2^ CFU/ml) were performed on filter paper and textiles as follows: *A. niger*—all materials; *Ch. globosum*—cotton, suede, and polyurethane; *T. reesei*—cotton, imitation suede, suede, polyester, and leather; *P. citrinum*—cotton and suede; *S. chartarum*—suede; *Cl. sphaerospermum*—cotton, suede, and leather.

Negative controls exhibited no visible or microscopic growth throughout the entire 6-week incubation period, confirming the absence of contamination. Conversely, growth controls displayed visible growth for all single strain and pooled mix inoculations as early as day two of incubation for 10^6^, 10^5^, and 10^4^ CFU/ml and as early as week one for 10^3^ and 10^2^ CFU/ml inoculum densities.

**Figure 2.**
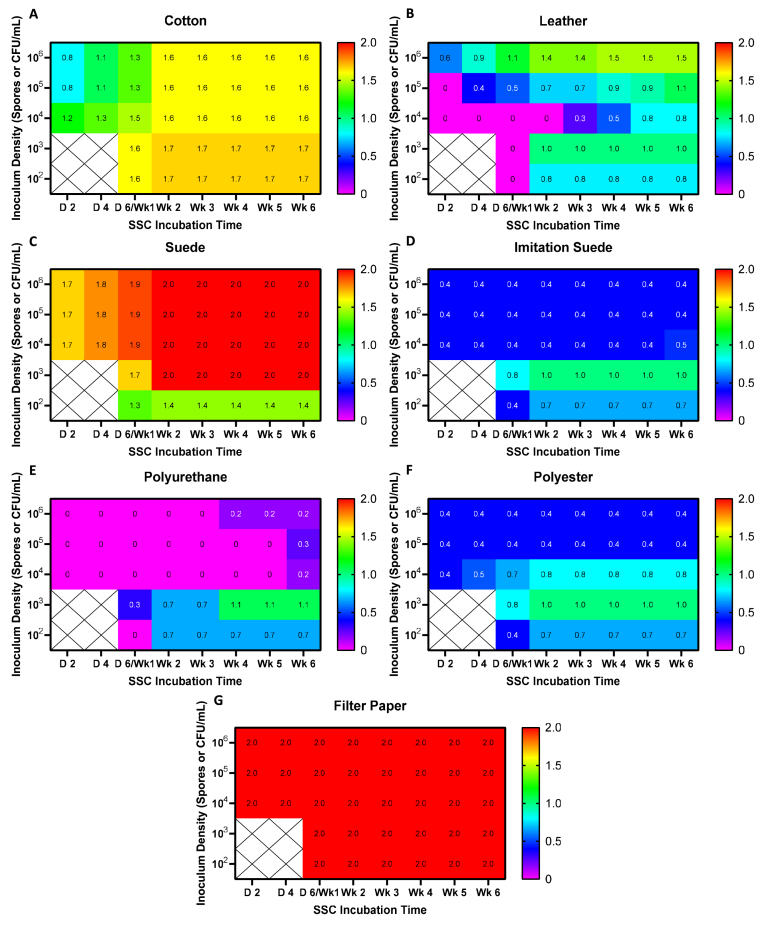
Textile susceptibility to single fungal strain inoculations under SSC. Heat maps show mean VMAS by textile material type of single fungal strain inoculations per textile material at 10^6^, 10^5^, 10^4^, 10^3^, and 10^2^ spores/ml throughout 6 weeks of incubation at 35°C ± 2°C at 65%–90% RH. VMAS was based on a grading scheme where 0 = no growth, 1 = microscopic growth, and 2 = macroscopic growth. Fungal strains tested include *A. niger, Ch. globosum, Cl. sphaerospermum, P. citrinum, S. chartarum*, and *T. reesei*. Textile materials tested include cotton (C), leather (L), polyester (P), polyurethane (PU), imitation suede (IS), and suede (S). Filter paper served as the growth control. Textile materials tested for *A. niger* at all inoculum densities include C, L, P, PU, IS, and S; for *Ch. globosum, Cl. sphaerospermum*, and *T. reesei* at 10^6^, 10^5^, and 10^4^ spores/ml include C, L, P, PU, IS, and S; for *P. citrinum* at 10^6^, 10^5^, and 10^4^ spores/ml include C, L, P, IS, S and at 10^3^, 10^2^ CFU/ml include C, S; for *Ch. globosum* at 10^3^, 10^2^ CFU/ml include C, S, PU; *Cl. sphaerospermum* at 10^3^, 10^2^ CFU/ml include C, L; for *T. reesei* at 10^3^, 10^2^ CFU/ml include C, L, IS, S, P; for *S. chartarum* at all inoculum densities include S only. Each textile material tested at every inoculum density for each strain was run in triplicates (*n* = 3).

**Figure 3.**
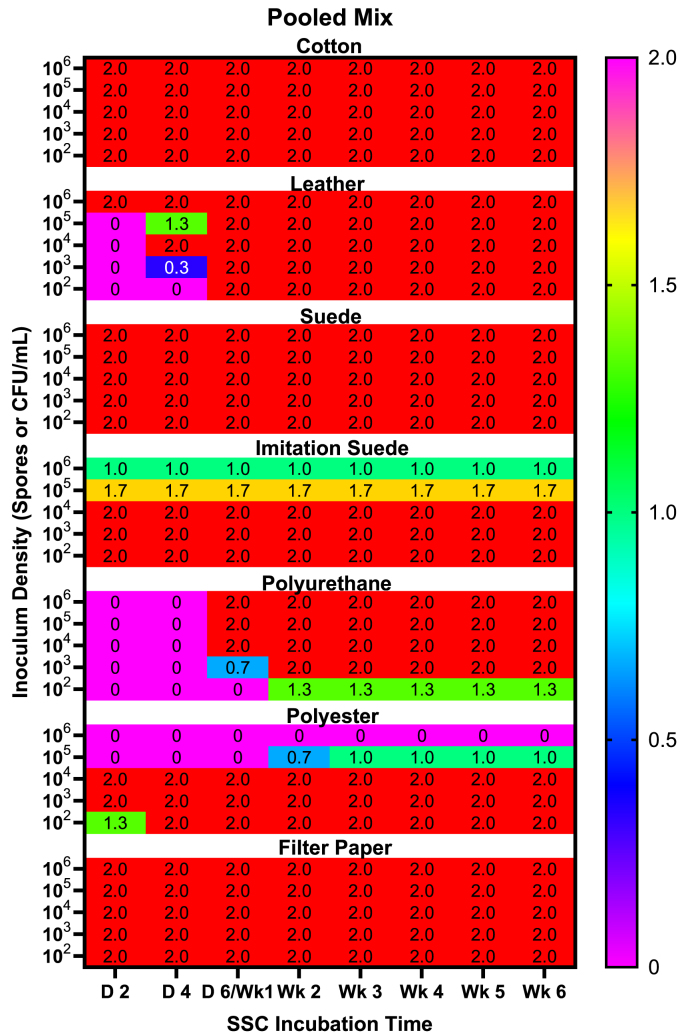
Textile susceptibility to pooled mix inoculations under SSC. Heat maps show mean VMAS by textile material type of the pooled mix inoculations per textile material at 10^6^, 10^5^, 10^4^, 10^3^, and 10^2^ spores/ml throughout 6 weeks of incubation at 35°C ± 2°C at 65%–90% RH. VMAS was based on a grading scheme where 0 = no growth, 1 = microscopic growth, and 2 = macroscopic growth. The pooled mix is composed of equal volumes of the corresponding inoculum densities of *A. niger, Ch. globosum, Cl. sphaerospermum, P. citrinum, S. chartarum*, and *T. reesei*. Filter paper served as the growth control. All textile materials were tested for the pooled mix at all inoculum densities. Each textile material tested at every inoculum density was run in triplicates (*n* = 3).

In the context of single fungal strain inoculations, distinct susceptibilities were noted among the various textiles (Fig. 2). Growth controls utilizing filter papers exhibited the highest vulnerability to mold growth across all inoculum densities (Fig. 2g). Cotton and suede emerged as the most susceptible textiles, exhibiting consistent and robust growth even at the lower fungal densities (10^2^ and 10^3^) (Fig. 2a, c). In contrast, imitation suede, polyurethane, and polyester demonstrated notable resistance, displaying limited susceptibility, especially at higher densities (10^5^ and 10^6^) (Fig. 2d–f). Notably, polyurethane demonstrated a higher level of resistance compared to imitation suede and polyester. Leather displayed susceptibility primarily at higher fungal densities and with prolonged incubation times (Fig. 2b). Notably, imitation suede consistently presented minimal growth across all fungal densities and incubation periods (Fig. 2d). The pooled mix inoculations (Fig. 3) generally aligned with the susceptibilities observed in single strain inoculations (Fig. 2), but the pooled mix tended to exhibit consistent growth patterns across all textiles and densities.

### A detailed examination of individual textiles incubated under SSC revealed the following (Figs S8–S14)

Cotton—Single strains: consistent and rapid growth observed from day 2 onward at higher fungal densities (10^4^, 10^5^, and 10^6^). At lower densities (10^2^ and 10^3^), growth started later (week 1) but increased steadily. Pooled mix: rapid and consistent growth was observed at all fungal densities, maintaining high susceptibility throughout.

Leather—Single strains: minimal growth was observed at higher densities (10^5^ and 10^6^), especially after the first week. At lower densities (10^2^, 10^3^, and 10^4^), there was visible growth, with a delayed onset. Pooled mix: similar pattern, but consistent growth observed at all densities.

Imitation Suede—Single strains: no growth was observed for *Ch. globosum, Cl. sphaerospermum*, and *P. citrinum*. Limited growth was observed for remaining fungal densities and incubation periods. Pooled Mix: minimal growth remained consistent across all conditions.

Suede—Single strains: robust growth even at lower fungal densities (10^2^ and 10^3^). Growth was consistently high throughout the incubation period. Pooled mix: maintained robust growth at all densities, remaining highly susceptible.

Polyurethane—Single strains: limited growth is observed, primarily at higher densities (10^5^ and 10^6^). Some delayed growth is seen at lower densities. Pooled Mix: similar limited growth pattern, especially at higher densities (10^5^ and 10^6^).

Polyester—Single strains: no growth was observed for *Cl. sphaerospermum* and *P. citrinum*. Limited growth was observed for the remaining fungal strains, especially at higher densities (10^5^, 10^6^). Pooled mix: consistent limited growth observed across all densities.

**Figure 4.**
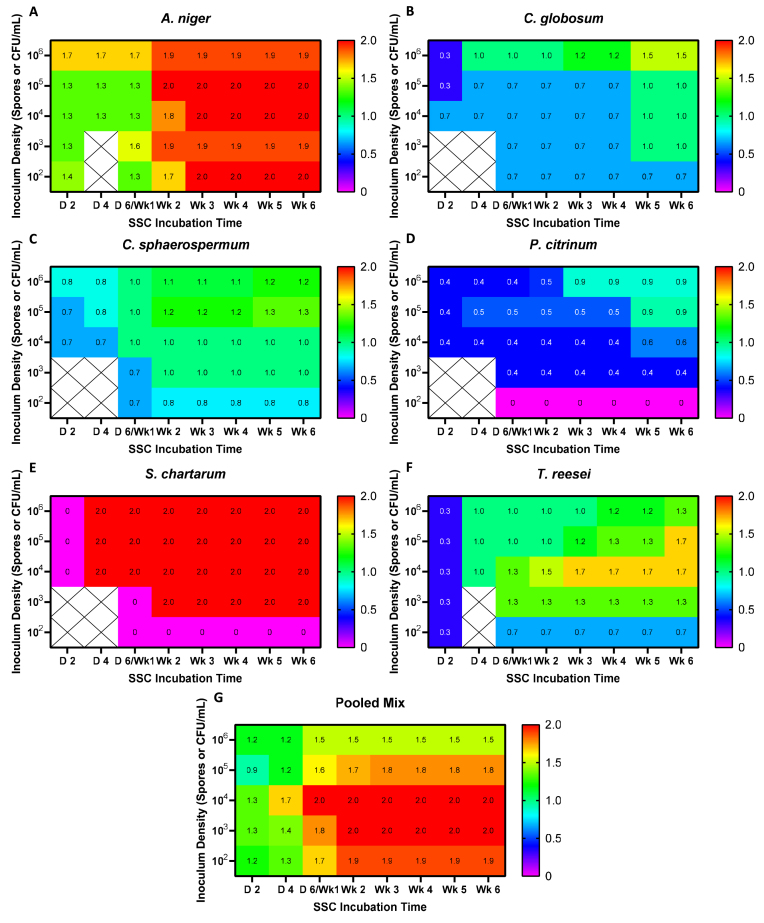
Fungal strain invasiveness under SSC. Each heat map shows the mean VMAS of all tested textiles for the indicated fungal strain. Fungal strains (or the pooled mix, as indicated) were inoculated at densities of 10^6^, 10^5^, 10^4^, 10^3^, and 10^2^ spores/mL throughout 6 weeks of incubation at 35°C ± 2°C at 65%–90% RH. VMAS was based on a grading scheme where 0 = no growth, 1 = microscopic growth, and 2 = macroscopic growth. Textile materials tested include cotton (C), leather (L), polyester (P), polyurethane (PU), imitation suede (IS), and suede (S). Filter paper served as the growth control. Textile materials tested for *A. niger* and the pooled mix at all inoculum densities include C, L, P, PU, IS, and S; for *Ch. globosum, Cl. sphaerospermum, T. reesei* at 10^6^, 10^5^, and 10^4^ spores/ml include C, L, P, PU, IS, and S; for *P. citrinum* at 10^6^, 10^5^, and 10^4^ spores/ml include C, L, P, IS, S and at 10^3^, 10^2^ CFU/ml include C, S; for *Ch. globosum* at 10^3^, 10^2^ CFU/ml include C, S, PU; *Cl. sphaerospermum* at 10^3^, 10^2^ CFU/ml include C, L; for *T. reesei* at 10^3^, 10^2^ CFU/ml include C, L, IS, S, and P; for *S. chartarum* at all inoculum densities include S only. Each textile material tested at every inoculum density for each strain was run in triplicates (*n* = 3).

In assessing the impact of different fungal strains on various textile materials (Fig. 4), distinct patterns emerged. *Aspergillus niger* exhibited robust growth, especially at higher densities (10^5^ and 10^6^), with consistent and rapid development observed from day 2 onward (Fig. 4a). *Chaetomium globosum* demonstrated limited growth across all densities, with some delay in lower-density (10^2^ and 10^3^) scenarios (Fig. 4b). *Cladosporium sphaerospermum* displayed moderate growth, remaining relatively unaffected by inoculum density variations (Fig. 4c). *Penicillium citrinum* exhibited limited growth at higher densities (10^5^ and 10^6^) (Fig. 4d). *Stachybotrys chartarum* showed robust growth on suede only at higher densities (10^4^ and above) but limited growth at lower densities (Fig. 4e). *Trichoderma reesei* displayed consistent growth at all densities, with higher densities (10^4^ and above) resulting in earlier and more rapid development (Fig. 4f). The pooled mix of all strains consistently demonstrated growth across densities with delayed growth onset and robust growth with elongated incubation (Fig. 4g).

## Discussion

It is widely acknowledged that textiles can foster the proliferation of bacteria and fungi, leading to the deterioration of natural fibers and, to a lesser extent, synthetic fibers (Sanders et al. 2021). The consequences of microbial growth on textiles encompass unpleasant odors, physical irritation, fabric decolorization, and the compromise of tensile strength (Szostak-Kotowa 2004, Gutarowska 2012). Microbial attachment to fabrics is influenced by the microbe type and the physiochemical properties of the fabric substrate. Factors such as substrate and microbial cell wall hydrophobicity play a role in microbial adherence, and the retention is observed to be time-dependent. Generally, a rougher surface tends to result in higher retention (Gupta 2007).

Natural fibers are more prone to microbial attacks, facilitated by their high moisture retention properties and accessible polymer linkages, particularly after fabric processing removes their protective layers (Gupta 2007). Moreover, natural fibers can serve as a source of nutrients and energy for microbes, offering carbohydrates or proteins (Gao 2008). The fabric’s characteristics, such as weave type, nonwoven or knitted structure, thickness, etc., also play a role in influencing moisture and heat retention properties, thereby influencing the habitat for resident microorganisms (Matusiak 2006, Premkumar 2016).

Unlike natural fibers, synthetic fibers often exhibit resistance to microbial attack due to challenges faced by microbial enzymes in breaking their carbon linkages, owing to their hydrophobic nature and limited adsorbing capacity (Shelley et al. 1953, Aminabhavi 1990, Zimniewska 2002, Pathak 2017, Siracusa 2019). Synthetic materials face biodeterioration primarily in the form of biofilm-related challenges, where microorganisms adhere to surfaces and create biofilms embedded in a polymer matrix. This process leads to various mechanisms that adversely affect the structure and function of synthetic polymeric materials, some of which include: coating surfaces, masking their properties and contaminating adjacent areas; increasing the release of additives and monomers through microbial degradation altering the polymer composition; enzymatic or radical attacks causing embrittlement and a loss of stability; swelling due to microbial filaments penetrating the polymer; and excretion of pigments by microorganisms leading to unwanted colors (Flemming 1998, Gu 2003).

In addition, synthetic fibers, including polypropylene, nylon, and polyester, can become vulnerable to microbial degradation when treated with finishing agents like polyethylene and polysiloxane emulsions. These additives facilitate microbial degradation by breaking down the polymer into manageable fragments using the acidic or basic byproducts of microbial metabolism, initiating a cycle of hydrolysis. This process renders even robust materials like polyurethane susceptible to breakdown under conducive conditions (Yau 1988).

Fabrics made from natural plant fibers, such as cotton, are widely used in clothing due to their hygienic properties. The relatively porous nature of cotton fibers allows for significant moisture absorption and retention (Shigaki 1993). These conditions create an environment conducive to mold growth, especially in humid environments. The surface texture of cotton fibers provides ample areas for moisture retention and settlement of mold spores. Untreated cotton lacks protective measures, enhancing its susceptibility to various molds. Fungal genera such as *Aspergillus, Alternaria, Cladosporium, Chaetomium, Fusarium, Myrothecium, Memnoniella, Penicillium, Stachybotrys, Trichoderma*, and *Verticillium* have been identified as the most active in cellulosic fabric degradation (Szostak-Kotowa 2004). Similar results were noted in this study where early growth was observed at very low inoculum spore densities for *A. niger, Ch. globosum, Cl. sphaerospermum*, and *T. reesei*, whereas slower growth was noted for *P. citrinum*. A short shipping duration is recommended, in part due to cotton’s susceptibility to visible mold growth under conducive conditions.

Similar vulnerability is noted in other fabrics from natural animal fibers, such as suede, typically made from the underside of animal hides, and leather, derived from animal hides (Fukushima 1975). Both fabrics can absorb moisture, contributing to their susceptibility to mold, especially in humid conditions. Suede exhibits a degree of porosity, allowing for moisture absorption; in addition, its textured surface may provide crevices for mold spores to settle, fostering growth. Polyamides found in leather, such as collagen or keratin, were previously found to have the lowest microbial resistance compared to synthetic polymers (Aminabhavi 1990, Gilon et al. 2023). The resistance to fungal growth and higher levels of minimal fungal density resulting in visible growth noted in this study could be attributed to chemical agents introduced in the tanning process to inhibit leather biodegradation (Nguyen 1891), as evidenced by the observed zones of inhibition suggesting the presence of leaching antimicrobials (Bruenke 2016). In addition, leather’s smoother surface compared to other natural fibers minimizes areas for mold spore settlement. Although a longer shipping duration may not result in visible mold growth, the variable porosity of leather, influenced by the tanning process (Harlan 1977), may render certain types more resistant to moisture absorption and in turn mold growth during transit. Wool has been considered to exhibit antibacterial properties due to its ability to reduce odor build-up from microbial activity. Interestingly, a study conducted by Ivankovic et al. (2022) showed that wool does not kill bacteria or inhibit their multiplication; instead, bacterial cells firmly attach to wool fibers forming a biofilm, resulting in a clear, no-growth zone when the wool specimen is lifted off the nutrient agar surface, removing the biomass and exhibiting an apparent antibacterial effect dependent on surface topography (Ivankovic et al. 2022).

Synthetic polymers tested, including polyester, polyurethane, and imitation suede, were also subject to some degree of biodegradation. The absence of mold growth on polyester and imitation suede for some strains in addition to the limited growth observed for others allows the flexibility of extended shipping durations due to their resistance to visible mold growth during transit. On the other hand, the limited and in some cases delayed mold growth on polyurethane allows for a longer shipping duration due to its moderate resistance to visible mold growth during transit.

Dominant fungal genera involved in polyurethane biodeterioration include *Cladosporium, Aspergillus, Penicillium, Chaetomium, Trichoderma*, and *Stachybotrys* (Szostak-Kotowa 2004). Except for *S. chartarum* and *P. citrinum*, all other fungal strains tested in this study showed a delayed onset of low levels of visible deterioration. A previous study by Darby et al. (1968) showed that polyether polyurethanes displayed varying degrees of resistance to fungal attack, ranging from moderate to high, whereas all tested polyester polyurethanes exhibited a high susceptibility. The susceptibility of polyethers was found to be correlated with the number of adjacent methylene groups in the polymer chain, with a minimum requirement of at least two adjacent methylene groups for a significant fungal attack. Additionally, the presence of side chains on the diol moiety of the polyurethane contributed to a reduction in susceptibility (Darby 1968).

Although *S. chartarum* only showed growth on suede in this study and *P. citrinum* showed no growth on polyurethane, it could simply be that the tested strains do not actively grow on these materials. Further testing can be carried out with other strains.

The delayed and sometimes more pronounced textile biodegradation observed with the pooled mix inoculations could be due to *P. citrinum* and *A. niger. Penicillium* and *Aspergillus* include species that can grow at much lower levels of moisture than other cellulolytic fungi. Under poor storage conditions, these less-demanding species can raise moisture levels, providing a conducive environment for more degradative species (Kowalik 1980).

The consolidated recommendations, outlining the anticipated minimum fungal densities leading to visible mold growth, derived from SSC incubations involving six examined fungal strains and a pooled mix at different densities on six distinct textile materials, are presented in Table 1. Two sets of recommendations were made based on different VMAS cutoffs. A recommendation was considered conservative when assessment scores were greater than one whereas a score greater than 1.5 was considered a liberal recommendation. The variability in minimum spore densities and time to visible damage highlights the diverse nature of fungal strains, each possessing unique mechanisms and proliferation times for causing damage to the textiles during shipping.

**Table 1.** Estimated minimum fungal densities until visible mold growth.

	Conservative (possible visible damage; VMAS > 1)		Liberal (likely visible damage; VMAS > 1.5)	
	Approx. minimum density (spores)	Shortest shipping duration	Approx. minimum density (spores)	Shortest shipping duration
**Fungal strain**				
*A. niger*	10^2^	2–6 days	10^2^	1–2 weeks
*Ch. globosum*	10^6^	2–3 weeks	10^6^	Up to 6 weeks
*Cl. sphaerospermum*	10^5^	1–2 weeks	10^6^	Up to 6 weeks
*P. citrinum* ^a^	10^6^	5–6 weeks	10^6^	Up to 6 weeks
*S. chartarum* ^b^	10^3^	1–2 weeks	10^3^	2 weeks
*T. reesei*	10^3^	4–7 days	10^4^	2–3 weeks
Pooled Mix	10^2^	2 days	10^2^	4–7 days
**Textile**				
Cotton	10^2^	2–7 days	10^2^	1 week
Leather	10^5^	5–6 weeks	10^6^	Up to 6 weeks
Suede	10^2^	2–7 days	10^3^	1 week
Imitation suede	10^6^	Up to 6 weeks	10^6^	Up to 6 weeks
Polyurethane	10^3^	3–4 weeks	10^6^	Up to 6 weeks
Polyester	10^6^	Up to 6 weeks	10^6^	Up to 6 weeks

a For all tested textiles except polyurethane.

b For suede only.

In cases where the contaminating fungal strain is known, *A. niger* and a mix of fungal strains were found to be the most invasive of the strains tested and were accordingly assigned the lowest minimum density with the shortest shipping duration. Ideally, materials contaminated with *A. niger* or a mix of fungal strains should be decontaminated or, if not possible, isolated before shipping. *Trichoderma reesei* demonstrated slightly less invasiveness and so was assigned a higher minimum density but within a similar shipping timeframe. Conversely, *Cl. sphaerospermum, Ch. globosum*, and *P. citrinum* demonstrated even less invasiveness, in order of highest to lowest, and so were assigned higher minimum densities with increasing shipping durations. *Stachybotrys chartarum* values were based on visible growth on suede only since no growth was observed with the other textiles tested.

In cases where the contaminating fungal strain is unknown, but the contaminated textile is known, cotton and suede were found to be the most susceptible materials to fungal contamination, being assigned the lowest minimum density with the shortest shipping duration. Ideally, these textiles should be decontaminated before shipping. Polyurethane showed moderate susceptibility and so was assigned a slightly higher minimum density but with the flexibility of a longer shipping duration. Finally, leather, polyester, and imitation suede were found to be the least susceptible of the tested textiles, allowing for much higher minimum densities and much longer shipping durations.

To detect the presence of mold and mold spores on consumer goods well before they are shipped and/or transported to their final location, it is essential to determine the minimum number of viable fungal cells required to cause visible damage. This study qualitatively and quantitatively compares mold growth severity of a range of inoculum densities of fungal species on different textile materials under SSC. Initial investigations were carried out where shipping conditions were simulated as realistically as possible under a best-case scenario wherein the textile alone is the contributing factor to any observed fungal growth or damage (i.e. no growth media was added). As the experiment progressed, pooled mix inoculums showed growth on polyester and rayon at higher inoculum densities; however, no growth or visible damage was observed on any textile for single-strain inoculations. Unfortunately, these results were not replicable with other textile raw materials which led to the development of a modified protocol based on TM30 of the AATCC Test III used to assess mold growth and evaluate textile specimens.

In this study where shipping conditions were simulated within a controlled laboratory environment, a significant contribution was made to understand fungal growth dynamics on textiles during transit. This approach aligns with the increasing necessity for research addressing microbial contamination risks associated with textile transportation. Notably, to the best of the authors’ knowledge, no studies have been carried out on mold growth progress throughout the shipping duration. These findings, assessed under SSC, provide insight into susceptibility patterns and practical implications for industries relying on textile transport in the distribution chain. This study not only sheds light on challenges introduced by microbial contamination during overseas container shipping but may also offer potential solutions and preventive strategies for minimizing these risks. By bridging the existing gap in knowledge, this research aims to empower industries to implement effective measures that enhance the resilience of textiles against microbial threats, ensuring the delivery of high-quality products to consumers worldwide.

While this study contributes valuable insights into the interplay of fungal strains, textile types, and inoculum densities under SSC, it is essential to acknowledge certain limitations, such as: (i) Mold growth on consumer goods could be the result of the molds’ ability to utilize the material as a carbon source or simply due to the materials being contaminated with a carbon source that supports mold growth. (ii) The possibility that providing mineral salts resulted in favorable conditions where mold utilizes the textiles themselves as a carbon source. Although mineral salts may be present on textiles as a result of pre- or post-treatments or due to environmental contamination, they may present in a wide range of concentrations that may inhibit or facilitate mold growth. (iii) Temperature and RH fluctuations were not considered, possibly resulting in more favorable growth than in real-world shipping conditions.

Despite these limitations, this study can serve as a crucial steppingstone and guide for manufacturing or shipping companies. The findings may particularly assist in the development of effective detection methods and the establishment of reliable mold limits, offering practical tools for industries seeking to enhance their understanding and control of fungal contamination risks in transit.

## Supplementary Material

qvae035_Supplemental_Files

## Data Availability

The data supporting this article are included within the article itself and in the supplementary material available online.
