# Supplementary material for: Assessing mold growth severity on various textiles: comparative analysis of inoculum densities and fungal strains
Source: Sustain Microbiol. 2024 Dec 18;2(1):qvae035. doi: 10.1093/sumbio/qvae035 (PMC13375309; doi:10.1093/sumbio/qvae035)
Supplement: Supplementary file 1 [file qvae035_Supplemental_Files.zip › Fig. S13.pdf]

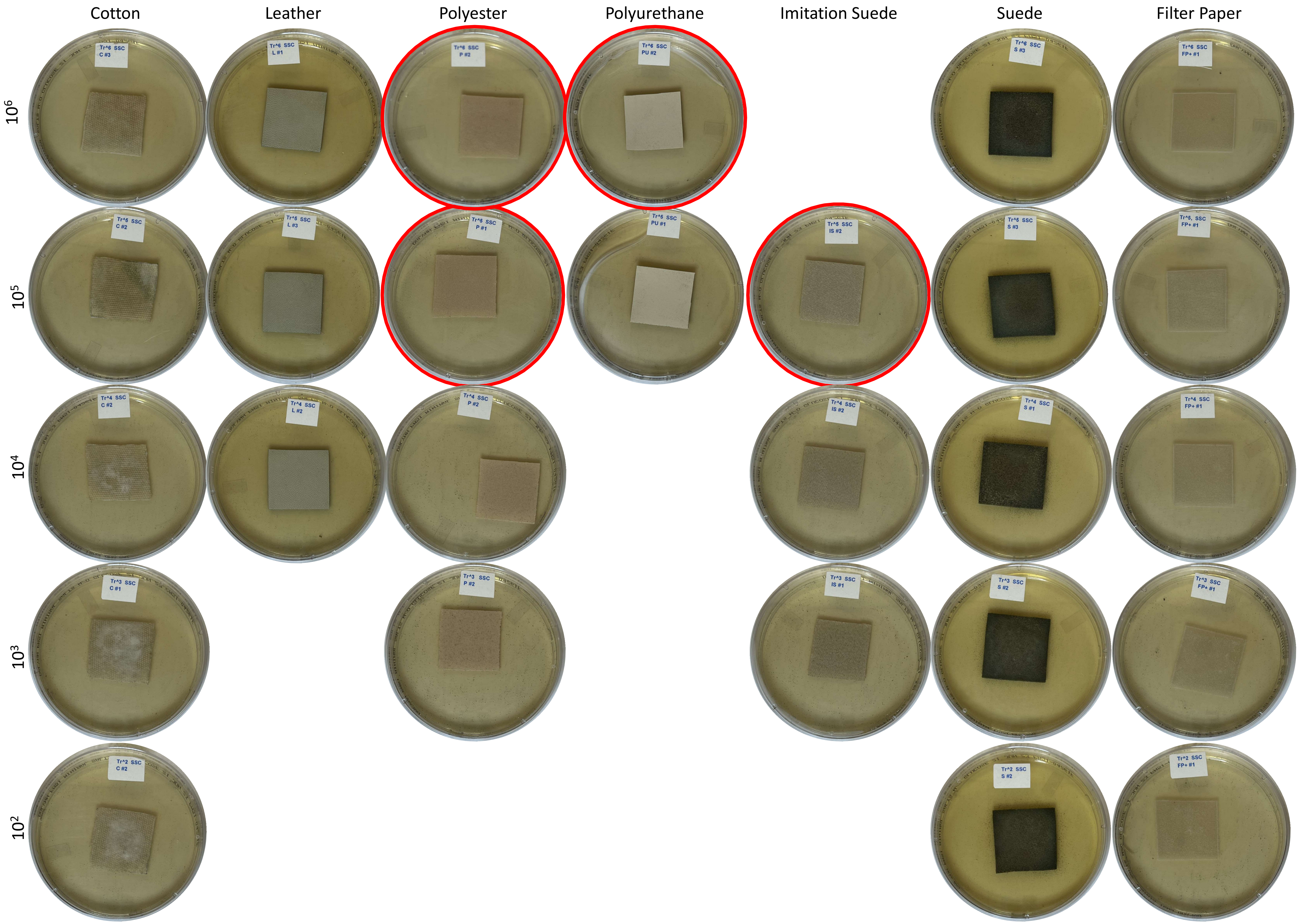
Cotton
Leather
Polyester
Polyurethane
Imitation Suede
Suede
Filter Paper
10^6
10^5
10^4
10^3
10^2
