# Supplementary material for: Assessing mold growth severity on various textiles: comparative analysis of inoculum densities and fungal strains
Source: Sustain Microbiol. 2024 Dec 18;2(1):qvae035. doi: 10.1093/sumbio/qvae035 (PMC13375309; doi:10.1093/sumbio/qvae035)

Mold in the Distribution Environment

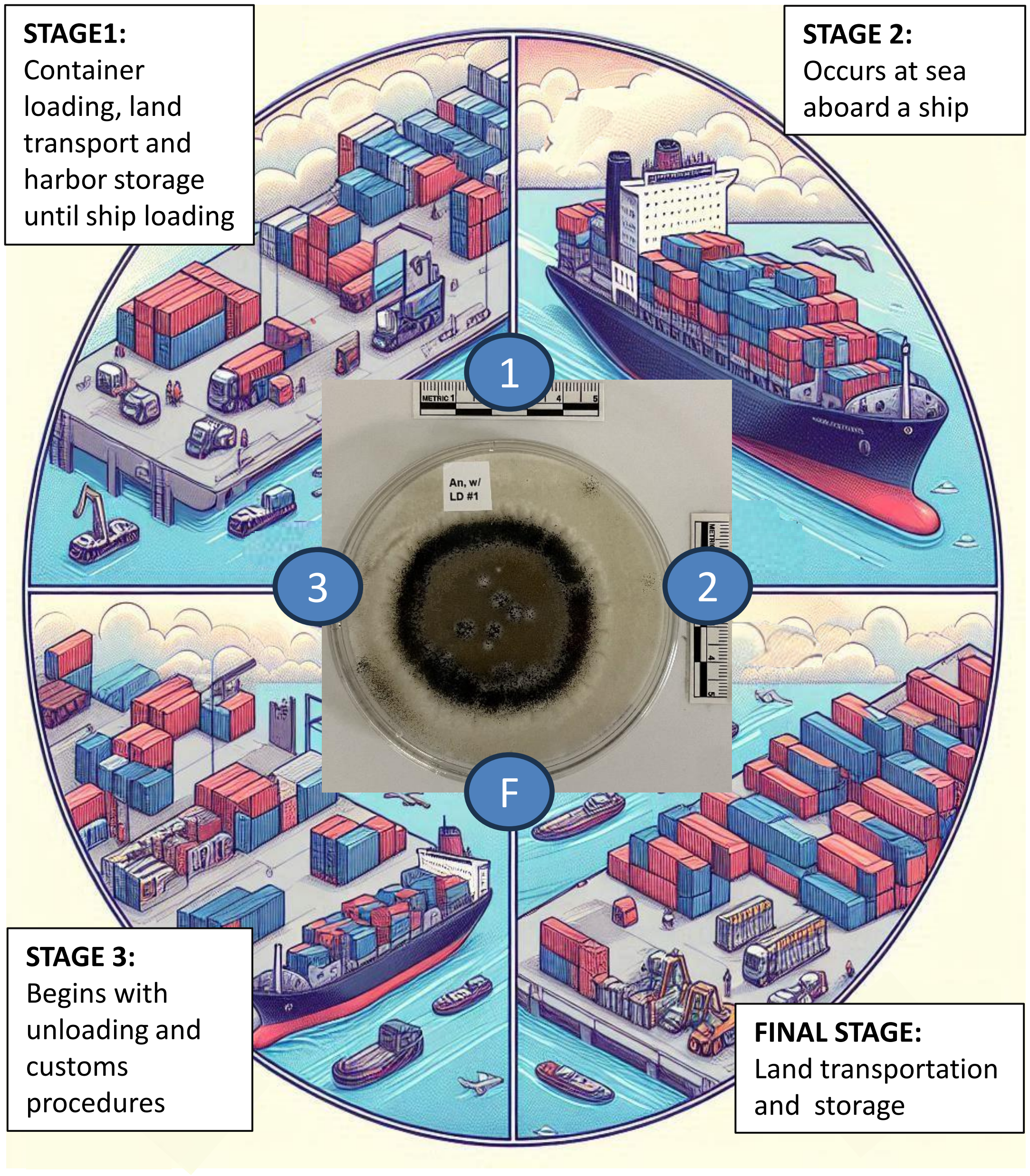

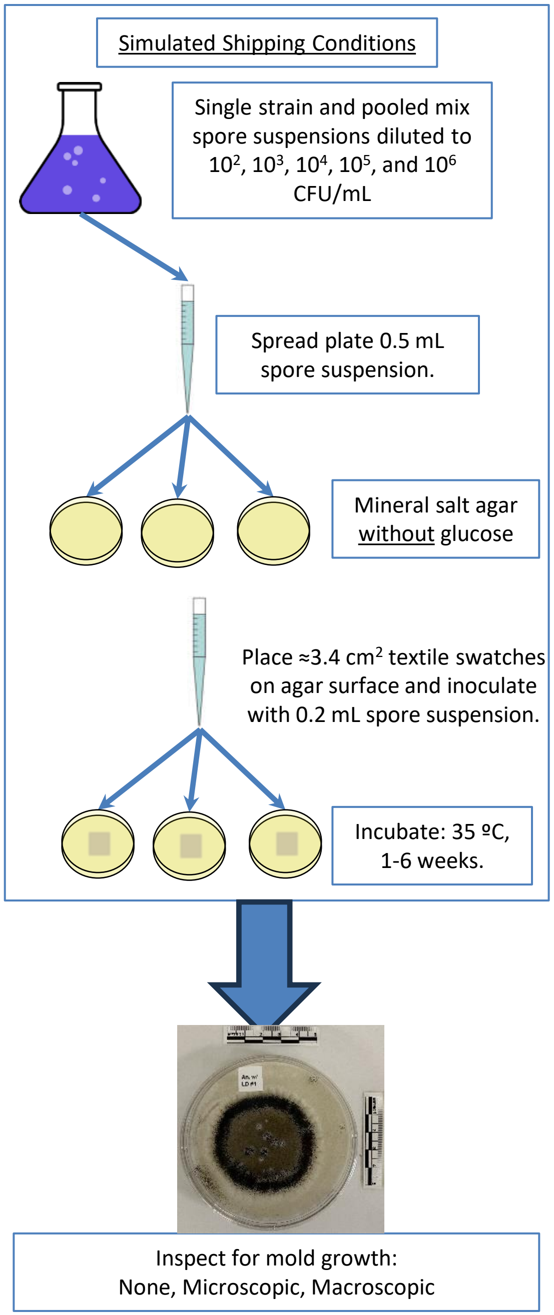

Supplement: Supplementary file 1 [file qvae035_Supplemental_Files.zip › Graphical Abstract.pdf]
